# Are we ready for a biological diagnosis of Parkinson’s Disease?

**DOI:** 10.1038/s41419-025-08076-z

**Published:** 2025-11-07

**Authors:** Angelo Tiziano Cimmino, Giulia Di Lazzaro, Antonio Pisani, Anna Rita Bentivoglio, Paolo Calabresi

**Affiliations:** 1 Department of Neurosciences, Università Cattolica del Sacro Cuore, Rome, Italy; 2 Neurology Unit, Fondazione Policlinico Universitario Agostino Gemelli IRCCS, Rome, Italy; 3 Department of Brain and Behavioral Sciences, University of Pavia, Pavia, Italy; 4 IRCCS Mondino Foundation, Pavia, Italy

**Keywords:** Diagnostic markers, Parkinson's disease

## Abstract

The diagnosis of Parkinson’s disease (PD) is currently based on clinical criteria, centered on the characteristic motor syndrome. However, motor manifestations become evident only after a significant proportion of nigro-striatal dopaminergic neurons have already undergone neurodegeneration. The recent “*NSD-ISS*” and “*SynNeurGe*” research frameworks have proposed new biological diagnostic criteria focusing on α-synucleinopathy, neurodegeneration, and genetic biomarkers, independent of clinical manifestations. These proposals intend to detect the disease at a “biologically early phase” to foster advances in research and development of disease-modifying treatments. While the shift to a biological approach is mandatory to better understand PD, challenges to the new frameworks remain, including inherent criticisms, limitations in explaining PD clinical-biological complexity, restricted clinical applicability and related ethical concerns. In this paper, we describe the historical path toward the two biological proposals, explore critical issues and knowledge gaps emerging from them, and discuss the risk of their premature application in the clinical setting.

## Introduction

Parkinson’s disease (PD) is currently diagnosed based on the clinical criteria proposed in 2015 by the International Parkinson and Movement Disorders Society (MDS) [1], which define the disease based on the combination of its characteristic clinical manifestations, with motor symptoms and signs playing the primary role. Nevertheless, PD motor features become clinically evident only when at least 60-80% of the substantia nigra pars compacta (SNpc) dopaminergic neurons are lost [2], as a result of a pathobiological process started many years earlier.

In the current view, PD overt clinical expression is preceded by a period of molecular and cellular events in the absence of clinical features (“preclinical PD”) or with mild motor and non-motor manifestations below the threshold for a formal diagnosis (“prodromal PD”).

This idea has strongly stimulated the identification of disease biomarkers for the early preclinical/prodromal stages, to seek possible disease-modifying therapies (DMTs) in this temporal window.

The novel possibility of in vivo identification of Lewy pathology (LP) through α-synuclein (α-syn) seed amplification assays (SAA) has represented a turning point and has provided the foundation for the recent proposal of two research frameworks aimed at establishing a neurobiological diagnosis of PD [3, 4].

These proposals arise as tools for research use only, intending to redefine PD relying only upon its biological hallmarks, independent of clinical manifestations. Such a shift in the PD-defining paradigm has been presented as an appropriate, timely, and necessary step to stimulate advances in basic and clinical research and developments in DMTs. Nevertheless, it conceals the risk that its premature broad extension could make the clinical diagnosis of PD unnecessary, in contrast to what has been done for more than two centuries [1, 5–8].

Like all paradigm shifts, the two proposals have provoked dissenting opinions and are producing stimulating discussions regarding criticisms and future challenges.

In this paper, we describe the historical and conceptual pathways that, over two centuries, from a purely clinical definition, have led to the frameworks for the biological definition of PD, then examine their pivotal ideas, the critical issues and the knowledge gaps emerging from them, to understand whether we are ready for a biological diagnosis of PD.

## The pathway towards the definition of diagnostic criteria

**Fig. 1:**
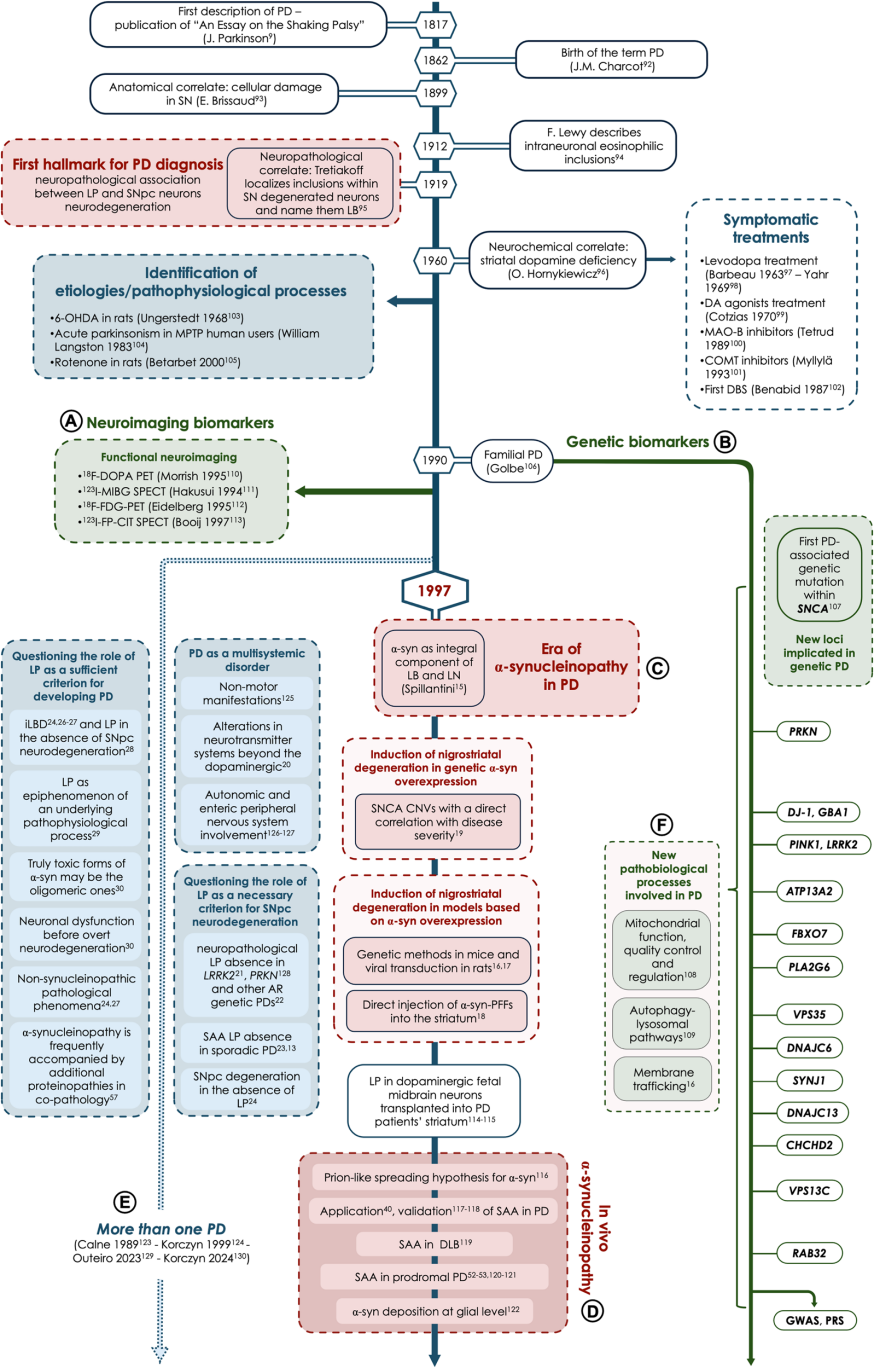
The milestones towards our understanding of PD. From the first formal description of the clinical phenotype by James Parkinson - later named PD by Charcot - to the early 20th century, progressive evidence has shed light on PD’s etiological, pathophysiological, biochemical, and neuropathological aspects. This progress culminated in the 1990s, with the great expansion of PD biomarkers, including the development of neuroimaging biomarkers (**A**) and the discovery of the first genetic forms of PD (**B**). The breakthrough moment in this history was the identification of α-syn as an integral component of LB and LN (**C**), marking the beginning of the “α-synucleinopathy era” in PD and “inextricably” linking the histories of α-syn and PD. The identification of SNCA CNVs, with a direct correlation with disease severity, and the induction of nigrostriatal degeneration in models based on α-syn overexpression, strongly supported α-syn role in PD pathogenesis. The subsequent evidence that aggregated pathological α-syn could propagate between neurons, inducing the aggregation and deposition of physiological α-syn isoforms, gave rise to the prion-like spreading hypothesis of α-syn, thus catalyzing the development of SAA to detect “in vivo” α-synucleinopathy (**D**). Alongside this “synthetic” strand (aimed at identifying in α-synucleinopathy the whole biological basis of PD), a parallel series of evidence has highlighted the clinical complexity and biological heterogeneity within PD (**E**). This evidence questioned the “unifying” definition, emphasizing instead the need to consider “more than one PD”, underscoring PD’s nature as a multisystem disorder and questioning the role of LP as a sufficient and necessary criterion for PD development. At the same time, the discovery of new genetic PD widened the scenario of the pathobiological processes involved (**F**). α-syn α-Synuclein, α-syn-PFFs α-Synuclein-preformed fibrils, AR autosomal recessive, CNV copy number variants, COMT catechol-O-methyltransferase, DA dopamine, DBS deep brain stimulation, iLBD incidental Lewy Body Disease, LB Lewy bodies, LN Lewy neurites, LP Lewy pathology, MAO-B monoamine oxidase B, MPTP 1-methyl-4-phenyl-1,2,3,6-tetrahydropyridine, PD Parkinson’s disease, PRS polygenic risk score, SAA seed amplification assay, SN substantia nigra, SNpc substantia nigra pars compacta, ^123^I-FP-CIT SPECT ^123^iodo-2β-carbomethoxy-3β-(4-iodophenyl)-N-(3-fluoropropyl) nortropane, ^123^I-MIBG SPECT ^123^iodo-metaiodobenzylguanidine single photon emission computed tomography, ^18^F-DOPA PET ^18^fluoro-dihydroxyphenylalanine positron emission tomography, ^18^F-FDG-PET ^18^fluoro-fluorodeoxyglucose positron emission tomography, 6-OHDA 6-hydroxy-dopamine.

Based on evidence regarding the complex etiopathogenetic and pathophysiological mechanisms at its basis, our understanding of PD has evolved (Fig. 1). In parallel, several diagnostic criteria have been progressively proposed.

A general PD definition is hampered by its clinical and biological complexity, and the evolution of PD diagnostic criteria has been driven by the need to find an “all-encompassing” definition that could capture such heterogeneity.

For more than a century after James Parkinson’s clinical description [9], no formal diagnostic criteria were available, and PD diagnosis relied entirely on the recognition of the characteristic motor syndrome [10]. Then, after the first identification of the neuropathological hallmarks in *post-mortem* PD brains, there was a shift to a “*clinical-neuropathological*” definition, as the association between the typical clinical syndrome and SNpc neurodegeneration linked to the nigrostriatal presence of LP [11, 12]. Since these findings could only be demonstrated in autopsy specimens, the first diagnostic criteria proposed were entirely clinically based [5, 6, 8], with the role of neuropathology as *post-mortem* confirmation [13, 14].

The 20^th^ century witnessed a progressively deeper understanding of the mechanisms underlying PD, with the identification of several pathophysiological processes at its basis. The breakthrough moment in this scenario was the identification of α-syn as an integral component of Lewy bodies (LB) [15], and the growing evidence supporting its pathogenic role when misfolded and aggregated [16–19], thus marking the beginning of the “α-synucleinopathy era” in PD [10].

**Fig. 2:**
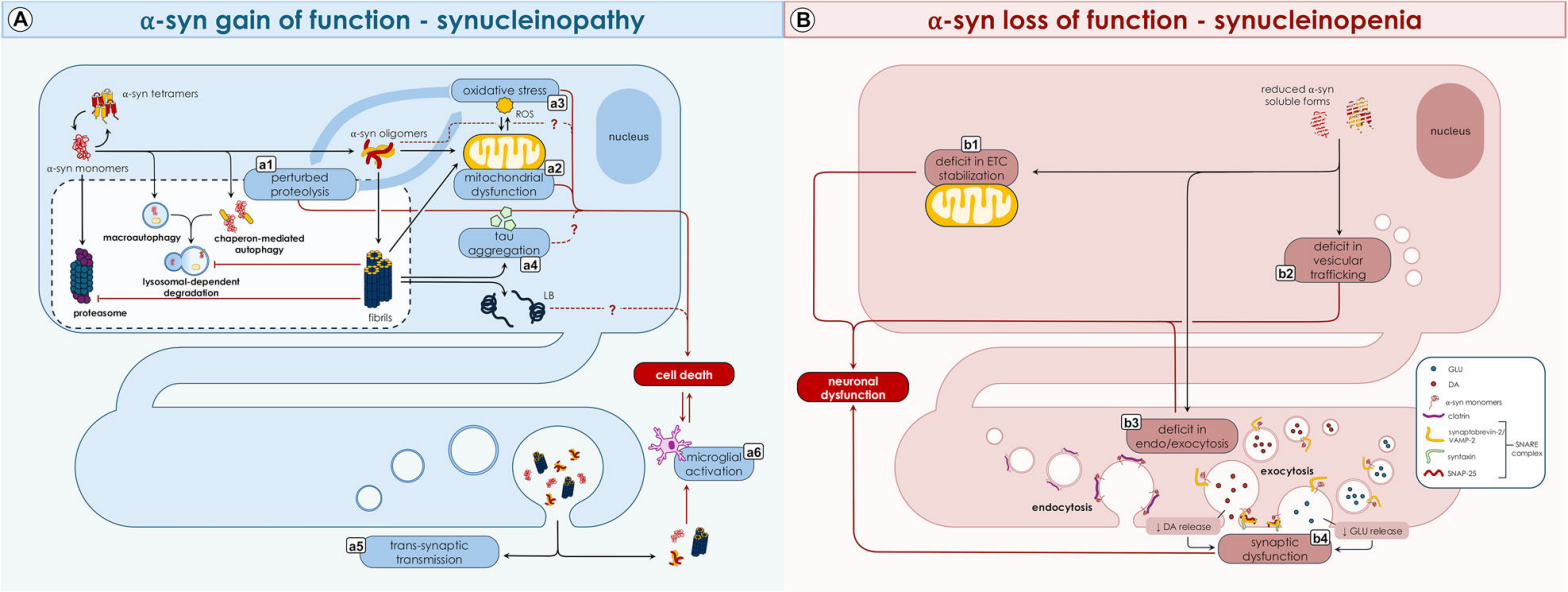
Gain of function (synucleinopathy) and loss of function (synucleinopenia) pathogenetic hypotheses for α-syn in PD. **A: Synucleinopathy Hypothesis (Gain of Function)**. The progressive aggregation of α-syn soluble forms (tetramers and monomers) into insoluble forms (oligomers and fibrils) leads to proteolysis disruption (**a1**), resulting in intracellular protein accumulation, and mitochondrial dysfunction (**a2**), which underlies the buildup of reactive oxygen species (ROS). Oxidative stress (**a3**) and defective proteolysis (**a1**) are thought to form a self-reinforcing pathological cycle, pivotal in driving cellular death and promoting the misfolding and aggregation of α-syn. A-syn aggregation process finally leads to the formation of intracellular LB and promotes the misfolding and deposition of other proteins, such as tau (**a4**), potentially contributing to cellular death. Moreover, evidence supports the hypothesis that misfolded and aggregated α-syn species can be transported extracellularly at the presynaptic terminal (**a5**), propagating to neighboring neuronal and glial cells. This intercellular transmission may trigger the activation of resident microglia (**a6**), further contributing to neurodegeneration in a bidirectional way. **B: Synucleinopenia Hypothesis (Loss of Function)**. The reduction of physiological (and functional) intraneuronal soluble α-syn forms disrupts several critical neuronal processes. These include deficits in the stabilization of the mitochondrial electron transport chain (ETC) (**b1**), resulting in mitochondrial dysfunction, and impairments in intracellular vesicle trafficking (**b2**). Reduction of functional α-syn also underpins presynaptic vesicle dynamics, where α-syn plays a crucial role in regulating endocytosis and exocytosis (**b3**). This results in the reduction of dopamine and glutamate presynaptic release, leading to synaptic dysfunction (**b4**). Collectively, these impairments contribute to α-syn-mediated neuronal dysfunction.

Simultaneously, the involvement of neurotransmitter systems beyond the dopaminergic [20], the neuropathological absence of LP in a fraction of genetic [21, 22] and sporadic [23–25] PD patients and the identification of LP in asymptomatic individuals (named *incidental Lewy Bodies Disease* - iLBD) [24, 26, 27] with no SNpc neurodegeneration [28] questioned the role of LP as a necessary and sufficient criterion for PD development. Altogether, these findings led to the alternative hypothesis that LP may represent an epiphenomenon of underlying pathophysiological processes [29] and that the truly toxic α-syn species may be the oligomeric forms [30], capable of inducing neuronal dysfunction prior to overt neurodegeneration [16, 30], or that other non-synucleinopathic pathological phenomena may be involved [24, 27, 29, 31–33] (Fig. 2).

Following the concerns regarding the role of LP as the “*arbiter of diagnosis*” [12, 14], in 2015 the MDS, while developing the new set of PD diagnostic criteria [1], explicitly chose to overcome its biological heterogeneity by basing the disease definition on the clinical syndrome, incorporating [^123^I]metaiodobenzylguanidine (MIBG) scintigraphy (among “*supportive criteria*”) and presynaptic dopaminergic system neuroimaging (among “*absolute exclusion criteria*”) as non-clinical biomarkers. This approach was maintained in the subsequent research criteria for “prodromal PD” [34], towards a conception of PD as a “*clinical-pathophysiological*” entity, diagnosable by the combination of clinical features and biomarkers of neurotransmitter systems’ pathophysiological alterations.

The recent growing body of PD biomarkers [35] - novel neuroimaging markers [36], new loci in genetic PD [37] - and the expanding field of molecular biomarkers, with in vivo demonstration of α-synucleinopathy through immunohistochemistry/immunofluorescence [38] and SAA [39, 40], paved the way for redefining PD based on its biological underpinnings. Thus, a further shift towards a “*biological*” perspective was made.

Consequently, in 2024, two separate study groups proposed two novel research frameworks - the “*neuronal synuclein disease integrated staging system*” (NSD-ISS) [3] and the “*SynNeurGe*” classification system [4] - redefining PD based on pure biology, freeing it from the clinical manifestations in the role of diagnostic definer.

Hence, the evolution of the concept of PD reached the present day undergoing a complete reversal: from a “*purely clinical*” entity, through a “*clinical-neuropathological*” phase, first, and a “*clinical-pathophysiological”* definition, then, arriving to the present in a “*purely biological”* redefinition [10].

## The proposals for the biological definition of PD

The proposal of the “*SynNeurGe*” classification system [4] and “*NSD-ISS*” [3] formalized the transition towards an entirely biological definition of PD.

Although these two frameworks differ in several aspects, they share their fundamental cornerstones.

Both proposals start from the observation that PD diagnosis is currently made after motor onset, many years after the pathobiological process has started. Consequently, the “early clinical” stage of PD is already an “advanced biological” stage, and this may have hindered the development of DMTs to date. The authors claim that a diagnosis purely relying on biological criteria would permit intercepting PD at a “biologically early” stage.

Both schemes argue that PD clinical manifestations are heterogeneous, involving both motor and non-motor domains, potentially overlapping with other disorders; therefore, a clinically driven diagnosis results in limited specificity. Conversely, the definition of PD based on a biological construct would allow for encompassing this heterogeneity by creating an “umbrella” definition including different clinical entities sharing the same underlying biology. This “all-encompassing” definition retains the name “*Parkinson’s Disease*” in the SynNeurGe system, while it is a new nosographic entity, “*Neuronal Synuclein Disease*” (NSD), in the NSD-ISS.

Both frameworks propose a new definition of PD based on three shared biological criteria: in vivo α-synucleinopathy, in vivo α-syn-associated neurodegeneration, and genetic contribution – independent of clinical manifestations. Both proposals are presented as research frameworks, with their applicability to routine clinical practice described as premature and currently inappropriate; both acknowledge the ethical implications and the need for prospective validation in future studies.

The main difference between the two proposals lies in the operationalization of the disease definition (Table 1) and their ultimate purpose. NSD-ISS is a progressive *“staging system”*, based on a “longitudinal-sequential” concept: the biological criteria permit the diagnosis and, when “integrated” with clinical manifestations, define a continuum of discrete disease “*stages*” that follow one another in a strictly sequential manner. In contrast, SynNeurGe is a “*classification system*”, based on a “transversal” approach, where the biological criteria allow for diagnosis and define biologically homogeneous subgroups among “sporadic” and “genetic” forms.

**Table 1:** Operational differences in defining the key biological diagnostic criteria between the *NSD-ISS* and *SynNeurGe* frameworks.

	*NSD-ISS*^3^	*SynNeurGe*^4^
**α-synucleinopathy**		
	Labeled as *Neuronal α-synuclein* (n-αsyn), defined by: ▪ CSF-α-syn-SAA	Labeled as *Parkinson’s type synucleinopathy* (or *Lewy type synucleinopathy*), defined by one of the following: ▪ CSF-α-syn-SAA ▪ Skin-α-syn-SAA ▪ Skin immunohistochemistry/immunohistofluorescence
*Distinction of neuronal α-syn from glial α-syn*	▪ CSF-α-syn-SAA alone is sufficient	Glial α-syn exclusion through one of the following criteria: ▪ CSF or plasma NfL elevation ▪ MSA-characteristic neuroimaging features
**Neuronal dysfunction or neurodegeneration**		
	Defined by: ▪ DaT-SPECT	Defined by one of the following: ▪ Dopaminergic PET or SPECT ▪ Metabolic FDG-PET ▪ Cardiac MIBG-SPECT
**Genotype**		
*Genotype alone is sufficient for diagnosis*	▪ Fully penetrant pathogenic variants in *SNCA*	▪ *G*_*F*_^*+*^ *status*, defined by fully penetrant genetic deleterious variants, as in *SNCA* (monoallelic triplications and SNVs) and *PRKN*, *PINK1*, and *PARK7* (biallelic SNVs and CNVs)
*Genotype alone is not sufficient for diagnosis*	▪ All other genotypes	▪ *G*_*P*_^*+*^ *status*, defined by deleterious variants with strong or intermediate predisposition, as in *SNCA* (monoallelic duplications), *LRRK2*, *GBA1*, *VPS35*, *CHCHD2* (monoallelic variants) ▪ *G*^*-*^ *status*, defined by polygenic risk scores or variants with low predisposition for PD or unknown genetic contributions

*CNVs* copy number variants, *DaT-SPECT* [^123^I]ioflupane-SPECT, *FDG-PET* [^18^F]fluorodeoxyglucose-PET, *MIBG-SPECT* [^123^I]metaiodobenzylguanidine-SPECT, *MSA* multiple system atrophy, *NfL* neurofilament light chain, *SNVs* single nucleotide variants, *α-syn-SAA* α-syn seed amplification assay.

In both frameworks, clinical manifestations are downgraded from the “disease-defining” role of the current PD diagnostic criteria [1] to staging elements, in NSD-ISS, or classification elements, in SynNeurGe.

## Challenges of the Biological Approach

The biological redefinition of PD and the consequent possibility of diagnosing PD based solely on biological criteria in asymptomatic patients represents a breakthrough event in the history of PD, paralleling what has been done recently in the field of Alzheimer’s disease (AD) [41]. Some have seen these proposals as a long-awaited step forward [42]; others, however, have highlighted challenges and limitations and the knowledge gaps they have raised [43–50].

### Criticisms of the biological definition proposals

The first challenge concerns the NSD-ISS and its longitudinal approach, which is the lack of sound evidence from prospective studies supporting a system based on sequential stages. The premise of NSD-ISS is that affected individuals progress through all stages of the NSD continuum. However, intrinsic contradictions are present, such as the transition from stage 1B (S^+^N^+^C^-^) to the subsequent stage 2A (S^+^N^-^C^+^), thus from a stage with positive evidence of dopaminergic dysfunction to a following stage with negative [^123^I]ioflupane-SPECT (DaT-SPECT). Beyond that, the logical leap is that this construct is based on prospective studies which only involve already symptomatic patients [51–53]. Formally, indeed, there are no prospective studies that identify the risk and rate of progression in asymptomatic α-synucleinopathy carriers without evidence of dopaminergic dysfunction (S^+^N^-^C^-^).

Furthermore, by defining NSD based on the necessary presence of both α-syn SAA in cerebrospinal fluid (CSF-aS-SAA) and DaT-SPECT positivity, clinically defined PD patients who exhibit nigrostriatal dopaminergic dysfunction but do not have α-synucleinopathy are excluded from the system. These include a proportion of monogenic PD with no evidence of LP, and sporadic PD with dopaminergic dysfunction but no α-syn in the SAA or neuropathological studies – these latter accounting for about 7% of the total [3, 23]. If, on the one hand, SynNeurGe, being capable of including genetic α-syn-negative PD cases, avoids this limitation, on the other hand, it fails to encompass sporadic PD patients without α-synucleinopathy (G^-^S^-^N^+^C^+^).

Moreover, neither proposal overcomes the problem of PD heterogeneity. In NDS-ISS, diverse clinical manifestations are classified together, being the expressions of a common biology. However, in this way, the boundaries between hitherto clinically distinct nosological entities (such as PD, Parkinson’s disease dementia, dementia with Lewy Bodies – DLB, isolated rapid eye movement sleep behavior disorder – iRBD, and hyposmia), become blurred, and these entities are traced back to the unifying biological foundation. SynNeurGe appears even more problematic concerning the role of clinical manifestations and the danger of an overly “broad” definition. Indeed, the authors correctly state that clinical manifestations can also occur in individuals without evidence of α-syn-related neurodegeneration since signs and symptoms may arise from neuronal dysfunction preceding overt neurodegeneration or from undetected neurodegeneration. This concept, though, also introduces the contradictory possibility that patients without a biological label for PD (i.e., G^-^S^+^N^-^, thus “*sporadic Parkinson’s type synucleinopathy*”, and G_P_^+^S^+^N^-^, thus “*genetic Parkinson’s type synucleinopathy*”), and therefore formally not affected, could be symptomatic. Moreover, in the definition of the “*neurodegenerative state*”, the SynNeurGe system includes MIBG-scintigraphy, a test with moderate specificity, as it can be positive in cardiologic diseases [43].

Furthermore, the attempt of both frameworks to merge the concept of PD with DLB, by extending the biological concepts that define PD to the latter, also appears problematic. DLB, in fact, doesn’t have the neuropathological identification of SNpc neurodegeneration as a required criterion. Therefore, DLB patients, especially in the early stages of MCI-DLB, exhibit low DaT-SPECT positivity [28, 47, 54]. Moreover, approximately 30% of clinically diagnosed DLB patients reported a CSF-aS-SAA-negative test in a recent large cohort [55]. Thus, the use of DaT-SPECT and aS-SAA as biological criteria to define DLB is insufficient, as a significant proportion of clinically defined prodromal and manifest DLB cases would not meet the proposed biological diagnostic criteria and would be excluded from clinical trials and research [50].

### Limitations in explaining clinical and biological complexity

The second challenge concerns the role given to the biological criteria, particularly to α-synucleinopathy, as the gold standard for PD diagnosis. In NSD-ISS, neuronal α-syn is both a necessary and sufficient criterion to define the entire NSD continuum. In contrast, SynNeurGe explicitly states that α-synucleinopathy is not necessary in PD definition, but it applies this concept only to the genetic forms, not to the sporadic, which remain bound to the demonstration of in vivo α-synucleinopathy.

Conceptually, all this can be traced back to the recognition of LP within degenerated nigrostriatal dopaminergic neurons in PD patients, and to the overly simplistic conceptual leap of equating pathology with pathogenesis [56]. This assumption established the dogma that LP is the hallmark of the neurodegenerative process in PD, and the subsequent identification of α-syn within LB suggested its pathogenic role.

However, α-synucleinopathy is neither a necessary nor sufficient element for PD.

It is not necessary, as in several monogenic (*LRRK2*, *PRKN*, and *PINK1)* and sporadic PD α-syn deposition is not detected in CSF-aS-SAA nor neuropathological findings, despite the presence of nigrostriatal dopaminergic dysfunction [3, 4, 23], which might be mediated by other pathological mechanisms [24]. Thus, SNpc neurodegeneration and LP may not be invariably pathologically linked [57]. In this context, it cannot be overlooked that clinical trials involving anti-α-synuclein strategies have thus far failed to demonstrate efficacy in slowing disease progression [49].

On the other hand, the identification of asymptomatic patients with LP (iLBD) in the absence of neurodegeneration undermines the role of α-syn deposition as the only pathological basis of the neurodegenerative process and, thus, as a sufficient criterion for PD [26]. While it could be thought that these individuals represent an “early biological stage” of PD not yet clinically manifest, these patients are older than the average age at onset of PD, suggesting that the relationship between PD and iLBD is more complex [24, 58]. In any case, to date, no sound evidence from prospective studies has demonstrated the role of α-syn deposition in asymptomatic patients as a progression marker towards PD [59].

The role of “disease state” marker attributed by both biological frameworks to SAA [50] appears particularly problematic also as SAA does not precisely reflect the in vivo process of α-synuclein aggregation: laboratory conditions under which aggregation occurs differ markedly from physiological environments, particularly in terms of α-synuclein concentration (*supersaturation*) [60]; additionally, the filaments produced in vitro exhibit structural differences from those observed in vivo, suggesting that the in vitro process does not fully recapitulate the in vivo mechanisms [59].

**Fig. 3:**
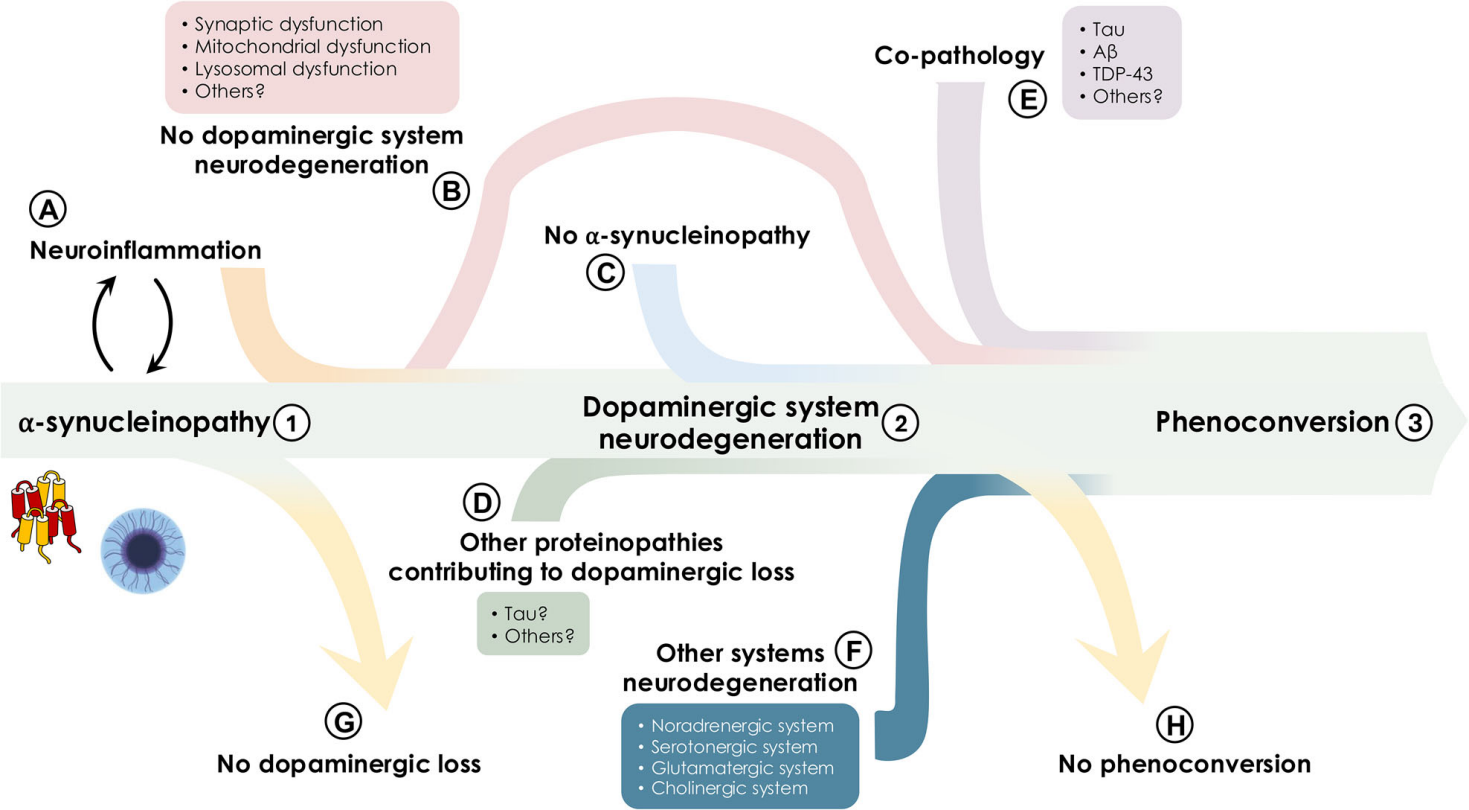
Potential alternative and complementary biological pathways in PD spectrum. The NSD-ISS is grounded in a longitudinal and sequential approach, wherein every affected individual progresses through all stages of the NSD continuum: from α-synucleinopathy [1], to subsequent nigrostriatal dopaminergic neurodegeneration [2], and eventually to the onset of clinical manifestations [3]. However, evidence suggests that additional events may play a role in the complex biological processes underlying PD, including: (**A**) neuroinflammation, particularly in the early stages of the disease, forming a vicious cycle with α-syn aggregation; (**B**) neuronal dysfunction in the absence of SNpc neurodegeneration, involving mechanisms such as α-syn-mediated synaptic, mitochondrial and lysosomal dysfunction, as supported by findings of patients with α-synucleinopathy and clinical manifestations without evidence of SNpc neurodegeneration; (**C**) α-synucleinopathy-independent processes, as observed in both genetic and sporadic forms of PD with SNpc neurodegeneration occurring in the absence of α-syn aggregation; (**D**) the involvement of other proteins, such as tau, which may contribute to the development of SNpc neurodegeneration; (**E**) co-pathology with other proteinopathies, suggesting interactions between multiple pathobiological processes associated to clinical manifestations; (**F**) other neurotransmitter systems neurodegeneration responsible for clinical manifestation, supporting the concept of PD as a “multisystem” disorder. Finally, the identification of individuals with iLBD, in the absence of SNpc neurodegeneration or clinical manifestations, raises the possibility that α-synucleinopathy alone may not necessarily lead to neurodegeneration and phenoconversion (**G**). Moreover, people with α-synucleinopathy and SNpc neurodegeneration may also not evolve towards overt phenoconversion (**H**).

Based on this evidence, it is clear that we currently understand PD as the association of a specific and recognizable clinical syndrome and a particular pathophysiological process represented by the progressive dysfunction and neurodegeneration of the nigrostriatal dopaminergic system [61]. This pathophysiological process would encompass multiple pathogenetic phenomena that ultimately lead to the dysfunction and neurodegeneration of the SNpc (Fig. 3).

Among these processes, tau protein may play a role, as it has been identified within LB [62] and its polymorphisms have been associated with increased risk of sporadic PD through genome-wide association studies [63, 64]. In non-synucleinopathic monogenic PD, such as *LRRK2*-related forms, tau pathology was observed [65, 66] and intracellular tau aggregates were identified even in some patients carrying α-syn variants [67, 68]. Moreover, the reduction in nigrostriatal dopaminergic neuron density is comparable between patients with “*mild motor deficits*” (MMD) [57, 69] associated with LP and MMD without nigral LP. However, both groups exhibit similar levels of phosphorylated tau aggregates, which are absent in the SNpc of age-matched controls, thus suggesting that nigrostriatal dopaminergic neurodegeneration may be a tau-mediated process independent of α-syn deposition.

Nonetheless, tau’s role in nigrostriatal neurodegeneration and its relationship with α-syn pathology – whether it precedes, follows, overlaps, or synergizes with it – remains an area not yet fully explored [57, 70–72].

Neuroinflammation may also be implicated in the pathogenesis of PD, as it is believed to contribute to the onset and propagation of neurodegeneration [73, 74]. In this process, α-synucleinopathy appears to play a central role, involving the activation of both innate and adaptive immunity. Misfolded α-synuclein isoforms can, in fact, activate resident microglia, causing the release of inflammatory cytokines [30, 75]. On the other hand, adaptive immunity also contributes, displaying specific reactivity towards α-synuclein [76, 77]. Neuroinflammation, in turn, creates a chronic pro-inflammatory environment that can spread to other brain regions, leading to increased oxidative stress and additional cellular damage, consequently triggering the release of damage-associated molecular patterns, further stimulating the inflammatory response. Neuroinflammation and neurodegeneration thus form a feed-forward loop that propagates, contributing to PD progression [78]. This process seems to play a primary role particularly in the early stages, where the pro-inflammatory Th1 profile predominates [79], with a tendency to decrease in the late stages, where Th2 and Th17 profiles become more dominant [80].

PD’s pathological question is made even more complex by the evidence that, within the same individual, different pathological actors can coexist in “co-pathology”.

In PD, in fact, each patient shows, on average, at least 3 pathologies, the most common being β-amyloid, tau, TDP-43 and vascular pathology [81]. While common thinking tends to associate a specific protein aggregate—proteinopathy—with a specific neurodegenerative disease, as a matter of fact, individual pathologies rarely exist in isolation [82]. The rule is that multiple pathological processes are simultaneously present, in various combinations, in a single neurodegenerative disease, in PD as well [83]. This is evident even considering that intraneuronal α-synucleinopathy is observed in atypical parkinsonisms [84] and in AD [85]. It thus becomes clear that α-synuclein cannot be regarded as the sole biological basis of PD but rather as a common feature of various neurodegenerative diseases and, more commonly, not the only one.

Nonetheless, the nature of the interaction between different proteinopathies – a simple overlap, a synergistic effect, or a more complex relationship – remains open, both in terms of pathophysiology and clinical implications [86].

The overall picture is further complicated by the fact that neurodegeneration in PD extends beyond the nigrostriatal dopaminergic pathway [20, 87, 88], affecting other central neurotransmitter systems (monoaminergic [89, 90] and cholinergic systems [91], among others). All of these contribute to the final phenotype and disease progression. Therefore, reducing the biological definition of PD to the nigrostriatal dopaminergic system offers only a partial view of the disease.

Both biological frameworks risk overlooking critical aspects of the complex pathobiology of PD by attributing it strictly to α-synucleinopathy. This overly reductive view may hinder research into alternative mechanisms and unduly constrain the development of DMTs by narrowing the focus and neglecting other potentially targetable pathogenetic processes [50]. The result of both frameworks appears to be the identification of a homogeneous subpopulation of patients in which the disease is marked by α-syn pathology, but that does not represent the entirety of PD individuals.

### Limitations in clinical applicability

Both frameworks are defined as research proposals and, at present, are not intended for routine clinical practice. Indeed, their clinical applicability, at least in the current form, appears inappropriate for several reasons.

The first reason is the limited global availability of the proposed biological diagnostic tools, which have a low diffusion compared to the high worldwide prevalence of the disease and are economically unsustainable for many countries. Consequently, the proportion of patients who can access a biological definition of the disease is low – even as a pure research tool, with a substantial concern regarding the potential reinforcement of global health disparities. In this regard, it is crucial to acknowledge that, although PD is a global disorder, the biological frameworks have been developed on data, particularly those related to SAA, from European and North American ancestry, thereby excluding underrepresented populations [59]. Finally, especially in the case of aS-SAA, the lack of standardization and reproducibility across centers significantly hampers its potential implementation at an international scale [59].

In parallel, the biological approach, with the possibility of defining an asymptomatic individual as “affected” raises ethical and healthcare concerns. First, since it labels as “affected” an individual who is currently healthy, although the likelihood, timing, and nature of any future clinical manifestation remain not predictable. Second, because patients with a clinical diagnosis of PD or DLB can be “stripped” of the disease label, as they do not meet the biological diagnostic criteria, with non-negligible implications for health care access.

The concept of disease is inherently linked to its clinical manifestation, and in this sense, despite being multifaceted, heterogeneous, and complex, PD originates as a clinically recognizable entity with specific clinical characteristics [44]. The risk is that the new biological criteria cause confusion by conflating disease with pathology (specifically α-synucleinopathy) and pathophysiology (the neurodegenerative dysfunction of the nigrostriatal dopaminergic system), although these are distinct concepts [49].

Although both frameworks are conceived as research proposals, there is a real danger of premature generalization into routine clinical practice, as already seen in the case of AD [43], with the risk of spreading ideas within the scientific community that are currently partial, require further validation, and are still plagued by significant knowledge gaps [59].

## Conclusions

The proposals of biological criteria for PD diagnosis represent a pivotal moment in the history of PD research, as they introduce biology as a “disease-defining” element for the first time.

This shift towards a biological approach in PD is mandatory, aiming at biological subtyping and stratification in such a clinically and biologically multifaceted entity. This approach is essential for a better understanding of the disease’s pathogenetic mechanisms, a more accurate definition of disease trajectories, and research into specific DMTs.

On the other hand, the current attempts at biologically redefining and diagnosing PD appear partial, preliminary, and, in some respects, contradictory. They are insufficient to explain the biological and clinical complexity of the disease, highlighting knowledge gaps in PD pathobiology that still need to be addressed (Table 2).

**Table 2:** Limitations and criticisms of *NSD-ISS* and *SynNeurGe* frameworks.

	*NSD-ISS*^3^	*SynNeurGe*^4^
**α-synucleinopathy (“*Pathology*”) defines “*Disease*”**	Yes	No
**“*Disease*” is possible without α-synucleinopathy (“*Pathology*”)**	No	• For sporadic forms → No • For genetic forms → Yes
**Inclusion of additional pathophysiological mechanisms (molecular pathways, neurotransmitter systems, co-pathology)**	No	No
**Clinical manifestations can precede nigrostriatal neurodegeneration**	No	Yes
**Linear progression from α-synucleinopathy to dopaminergic nigrostriatal degeneration, then to clinical manifestations**	Yes	No
**Merging of prodromal diseases, overt PD and overt DLB**	Yes	Yes
**Limited cost, global availability and worldwide standardization of the biological diagnostic tools**	Yes	Yes
**Ethical issues due to contradiction between biological and clinical diagnostic criteria**	Yes	Yes

**Fig. 4:**
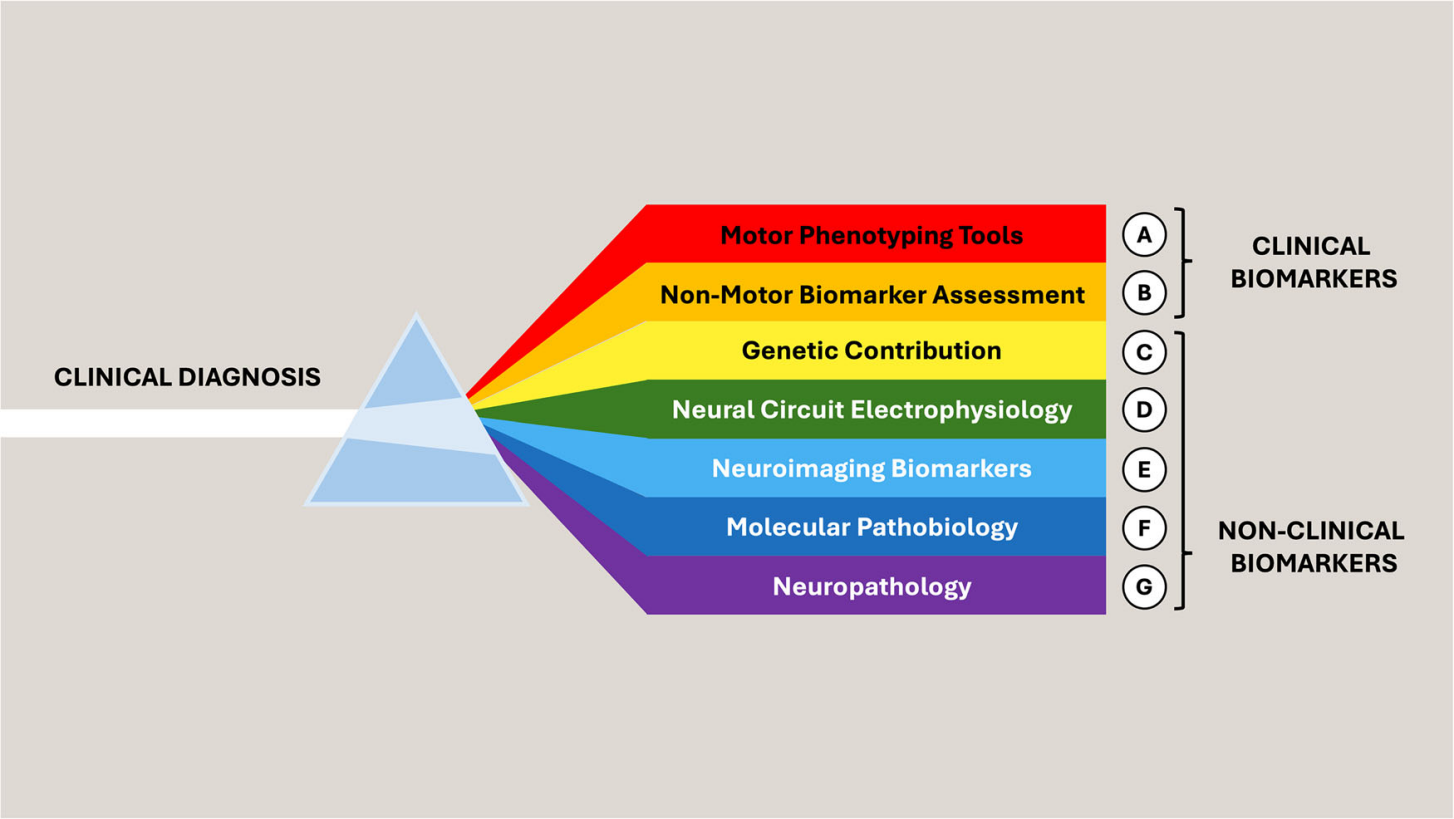
Towards a biological redefinition of PD: an integrative framework for deep subtyping. While the diagnosis of PD remains fundamentally clinical, a deeper understanding of disease heterogeneity requires the incorporation of diverse investigational domains, integrating multiple clinical and non-clinical biomarkers: (**A**) motor phenotyping tools, including kinematic analysis and wearable technologies, to allow out-of-office monitoring of motor clinical aspects; (**B**) deep evaluation of non-motor domains through instrumental testing of autonomic, sleep-related, and sensory functions; (**C**) genetic contributions, encompassing both monogenic forms and polygenic risk factors; (**D**) electrophysiological investigation of neural circuits involved in both motor and non-motor domains; (**E**) structural and functional neuroimaging to investigate disruptions across multiple neurotransmitter systems; (**F**) molecular and cellular pathobiology, focusing on key pathogenetic mechanisms such as neuroinflammation, proteostasis impairment, lysosomal and mitochondrial dysfunction, trafficking and endocytosis impairment, endoplasmic reticulum stress, and synaptic dysfunction; (**G**) in vivo and ex vivo neuropathology, including molecular techniques for the detection of specific proteinopathies such as α-synucleinucleinopathy. Together, these dimensions enable a deeper subtyping of PD and related disorders (including prodromal PD and DLB), filling current knowledge gaps and fostering the development of biology-based definitions, prognostic models, and ultimately DMTs.

The ultimate result of the biological proposals is the identification of a subgroup of individuals who share the common pathological finding of α-synucleinopathy – thus “*homogeneous for pathology*” – which constitutes a subset of the total PD patient population. Hence, biology becomes one of the tools available for deep subtyping, serving as a complement to the clinical diagnosis. It is in this direction that we propose the concept of “biological redefinition” should evolve: to maintain PD (as well as its prodromal forms and DLB) as a clinical syndrome, while integrating both clinical and non-clinical markers to enable a more refined stratification (Fig. 4). Only through this integrative approach can we begin to identify meaningful disease subgroups in PD (and DLB), the study of which will allow a more complete understanding of the underlying etiological, pathogenetic and pathophysiological mechanisms, ultimately paving the way for biology-based frameworks of definition, staging, prognostic trajectory mapping, and DMTs development.

Although still in their infancy, these two proposals have the great merit of paving the way toward a new scenario in PD history. But, if the question is “are we ready for a biological diagnosis of PD?”, our answer would be “maybe not”.

## Data Availability

Data sharing not applicable to this article as no datasets were generated or analyzed during the current study.
